# Efficacy of an HSP90 inhibitor, ganetespib, in preclinical thyroid cancer models

**DOI:** 10.18632/oncotarget.17180

**Published:** 2017-04-18

**Authors:** Shu-Fu Lin, Jen-Der Lin, Chuen Hsueh, Ting-Chao Chou, Chun-Nan Yeh, Ming-Huang Chen, Richard J. Wong

**Affiliations:** ^1^ Department of Internal Medicine, Chang Gung Memorial Hospital, Chang Gung University, Taoyuan, Taiwan; ^2^ Department of Pathology, Chang Gung Memorial Hospital, Taoyuan, Taiwan; ^3^ Laboratory of Preclinical Pharmacology Core, Memorial Sloan-Kettering Cancer Center, New York, NY, USA; ^4^ Department of Surgery, Chang Gung Memorial Hospital, Taoyuan, Taiwan; ^5^ Department of Internal Medicine, Taipei Veterans General Hospital, Taipei, Taiwan; ^6^ Department of Surgery, Memorial Sloan-Kettering Cancer Center, New York, NY, USA; ^7^ Current address: PD Science, Inc., Paramus, NJ, USA

**Keywords:** HSP90 inhibitor, ganetespib, thyroid cancer

## Abstract

Heat shock protein 90 is a molecular chaperon that maintains the correct folding and function of multiple client proteins. The inhibition of heat shock protein 90, which leads to the simultaneous degradation of multiple proteins involved in oncogenic signaling pathways, has revealed an innovative strategy to treat a variety of cancer types. We evaluated the therapeutic effects of ganetespib, a heat shock protein 90 inhibitor, in treating thyroid cancer. Ganetespib effectively inhibited cell proliferation in a dose-dependent manner in eight cell lines originating from four major histologic types of thyroid cancer (papillary, follicular, anaplastic and medullary). Ganetespib decreased cyclin-dependent kinase 1 and arrested cell cycle progression in G2/M phase. The expression of proteins involved in RAS/RAF/ERK and PI3K/AKT/mTOR signaling pathways was also inhibited. The RET level was decreased in a medullary thyroid cancer cell line. Ganetespib increased Bim expression, activated caspase-3 and induced apoptosis. *In vivo*, ganetespib retarded the tumor growth of anaplastic and medullary thyroid cancer xenografts with acceptable safety profiles. These findings indicate that ganetespib has potential in the treatment of patients with thyroid cancer.

## INTRODUCTION

Thyroid cancer is the most common endocrine malignancy and its incidence has increased in recent decades [1]. The major histologic types of thyroid cancer include well-differentiated cancer (papillary and follicular), anaplastic cancer and medullary cancer. These four types of thyroid cancer originate from follicular cells (papillary, follicular and anaplastic cancer) and parafollicular C cells (medullary cancer). The prognosis of well-differentiated thyroid cancer is usually favorable. However, the survival in patients who develop radioiodine-refractory distant metastases was only < 3-5 years [2]. Two multikinase inhibitors, sorafenib and lenvatinib, have been approved by the United States Food and Drug Administration for the treatment of radioiodine-refractory differentiated thyroid cancer. However, many patients receiving these agents develop progression disease and need additional therapies [3, 4]. Anaplastic thyroid cancer (ATC) is a rare and usually fatal thyroid malignancy, with a median survival of approximately 6 months. Medullary thyroid cancer (MTC) accounts for about 1.4-4% of thyroid malignancies. Two kinase inhibitors, cabozantinib and vandetanib improve progression free survival in patients with MTC. Nevertheless, both drugs are associated with toxic effects that usually lead to termination of treatment [5, 6]. Novel therapies with different therapeutic mechanisms are needed to improve the outcomes of patients with refractory thyroid cancer.

Heat shock protein 90 (HSP90) is the most abundant intracellular protein that is essential to maintain proper folding and maturation of proteins in mammalian cells [7]. Many client proteins of HSP90 are involved in cell cycle progression and cell survival. Inhibition of HSP90 results in the degradation of client proteins and leads to cell cycle arrest and apoptosis [8, 9]. Although ubiquitous in expression, HSP90 in tumor cells has an approximately 100-fold increased sensitivity to HSP90 inhibitors as compared with normal cells, demonstrating that HSP90 is a reasonable therapeutic target of cancer [10].

Ganetespib is a second-generation HSP90 inhibitor that exhibits potent cytotoxicity *in vitro* and demonstrates antitumor activity with promising safety profiles *in vivo* for a diverse group of cancers [11–17]. This drug has also shown promising effects against human breast and lung cancers in clinical trials [17, 18]. In this study, we explored the therapeutic efficacy of ganetespib in the treatment of thyroid cancer.

## RESULTS

### Cytotoxicity of ganetespib

**Figure 1:**
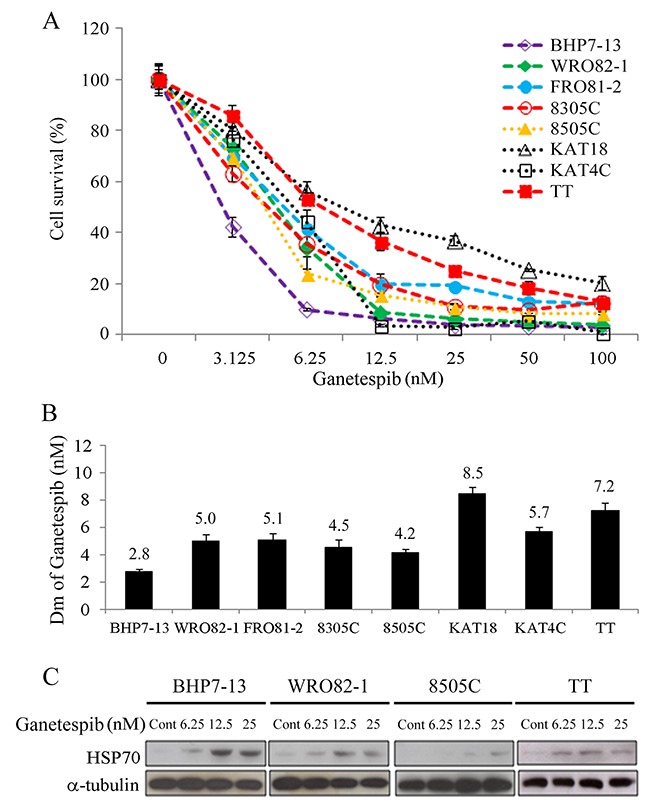
Ganetespib induces cytotoxicity and increases HSP70 expression in thyroid cancer cells **(A)** Cytotoxicity was studied in cells treated with a series of six 1:1 dilutions of ganetespib. Dose-response curves were obtained on day 4 by using LDH assay in eight thyroid cancer cell lines. **(B)** Median effect doses (Dm) of ganetespib on day 4 were calculated using CompuSyn software for each cell line. **(C)** The expression levels of HSP70 were evaluated using immunoblot in cells treated with ganetespib at indicated doses for 48 hours in BHP7-13, WRO82-1, 8505C and TT cells.

Ganetespib inhibited cell proliferation in eight thyroid cancer lines in a dose-dependent manner (Figure 1A). A low dose of ganetespib (6.25 nmol/L) impeded at least 43% of cell growth in all cell lines on day 4. Ganetespib at 100 nmol/L arrested > 95.7% cell growth in the well-differentiated thyroid cancer lines (BHP7-13 and WRO82-1), 87.7% in a follicular undifferentiated thyroid cancer line (FRO81-2), > 79.5% in the ATC lines (8305C, 8505C, KAT18 and KAT4C) and 87.0% in the MTC line (TT). The median-effect dose (Dm) of ganetespib on day 4 was calculated for each cell line (Figure 1B). The well-differentiated papillary thyroid cancer (BHP7-13) was the most sensitive cell line (Dm = 2.8 ± 0.2 nmol/L). An ATC cell line (KAT18) and the MTC line (TT) were the most resistant cell lines (Dm = 8.5 ± 0.5 nmol/L and 7.2 ± 0.6 nmol/L, respectively). The other cell lines demonstrated similar sensitivity (Dm between 4.2 ± 0.2 nmol/L and 5.7 ± 0.4 nmol/L).

The effects of ganetespib on the expression of heat shock protein 70 (HSP70) as a marker of HSP90 inhibition were evaluated at 48 hours in four cell lines representing papillary (BHP7-13), follicular (WRO82-1), anaplastic (8505C) and medullary (TT) thyroid cancer (Figure 1C). A low dose of ganetespib (6.25 nmol/L) increased HSP70 levels in BHP7-13 and TT. High doses of ganetespib (12.5 and 25 nmol/L) induced HSP70 expression in all cell lines. These data are consistent with prior reports noting that inhibition of HSP90 increases HSP70 expression [12, 17].

### Effects of ganetespib on cell cycles

**Figure 2:**
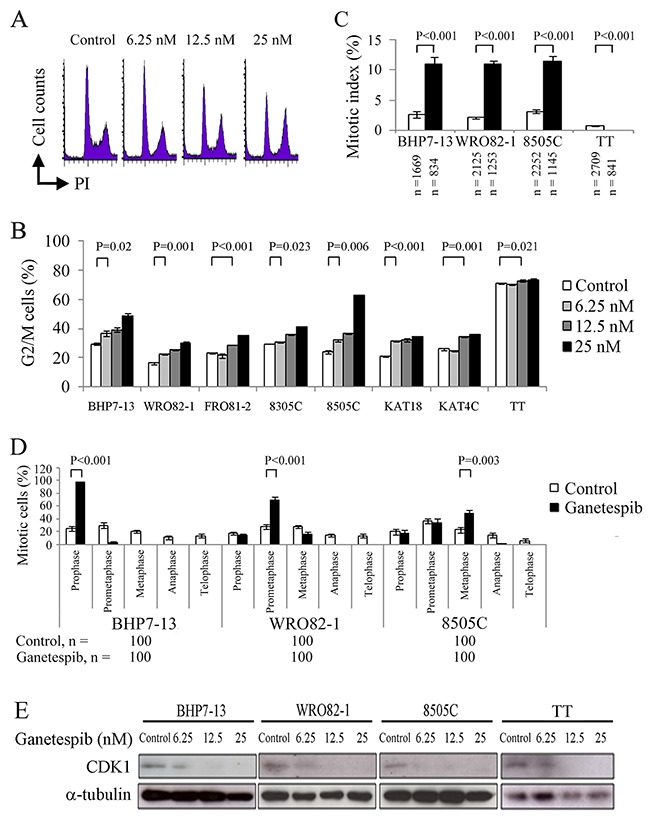
Ganetespib decreases CDK1 expression and inhibits cell cycle progression in G2/M phase **(A)** Analysis of the cell cycle by measurement of DNA content in BHP7-13 cells treated with ganetespib for 24 hours showed that increasing doses of ganetespib accumulated more cells in G2/M phase. **(B)** Statistical analyses of cell cycle data revealed dose-dependent effects of ganetespib to accumulate cells in G2/M phase at 24 hours (BHP7-13, WRO82-1, FRO81-2, 8305C, 8505C, KAT18 and KAT4C) and 48 hours (TT). **(C)** The percentage of thyroid cancer cells in mitosis was assessed after treatment with placebo or ganetespib (25 nmol/L) for 24 hours (BHP7-13, WRO82-1 and 8505C) and 48 hours (TT). Cells were stained with DAPI and α-tubulin and chromosome features were evaluated using immunofluorescence confocal microscopy. Mitotic index was assessed with a minimum of 834 cells counted from at least ten different fields for each condition. Ganetespib significantly increased the proportion of cells in mitosis in BHP7-13, WRO82-1 and 8505C cells. In contrast, ganetespib decreased the proportion of mitotic cells in TT. **(D)** The distribution of cells in mitosis was determined by counting 100 mitotic cells by confocal microscopy for each condition. Statistical analyses revealed mitotic cells accumulated in prophase (BHP7-13), prometaphase (WRO82-1) or metaphase (8505C) by the treatment of ganetespib (25 nmol/L) for 24 hours. **(E)** The expression level of CDK1 was evaluated using immunoblot in cells treated with ganetespib for 24 h. A low dose of ganetespib (6.25 nmol/L) was able to reduce CDK1 in BHP7-13, WRO82-1 and 8505C cells. High doses of ganetespib (12.5 and 25 nmol/L) decreased CDK1 expression in four cell lines.

Thyroid cancer cell lines were exposed to ganetespib at clinically relevant doses (≤ 25 nmol/L) for 48 hours (TT) or 24 hours (other cell lines) and the effect of ganetespib on cell cycle progression was evaluated [19]. A represented cell line, BHP7-13, revealed higher doses of ganetespib arrested more cells in G2/M phase (Figure 2A). Cell cycle data were analyzed for eight cell lines (Figure 2B). Compared with control cells, a low dose of ganetespib (6.25 nmol/L) induced cell accumulation in G2/M phase in 5 cell lines (BHP7-13, WRO82-1, 8305C, 8505C and KAT18). High doses of ganetespib (12.5 and 25 nmol/L) significantly increased the proportion of cells in G2/M phase for all cell lines. These data reveal ganetespib is able to arrest cell cycle progression in G2/M phase in thyroid cancer cells.

The ability of ganetespib to arrest cells in the mitotic phase was evaluated using confocal fluorescence microscopy (Supplementary Figure 1). Mitotic cells were identified and the mitotic index was calculated for four thyroid cancer cell lines (Figure 2C). Compared with control cells, ganetespib (25 nmol/L) treatment for 24 hours significantly increased the percentage of mitotic cells in BHP7-13 (10.9 ± 1.3% and 2.7 ± 0.5%, P < 0.001), WRO82-1 (10.9 ± 0.5% and 2.2 ± 0.2%, P < 0.001) and 8505C (11.4 ± 0.8% and 3.2 ± 0.2%, P < 0.001), demonstrating that ganetespib arrested cells in mitosis in these cell lines. However, in TT cells, ganetespib therapy for 48 hours significantly decreased the proportion of mitotic cells than control cells (0 ± 0% and 0.8 ± 0.1%, P < 0.001), revealing a block of mitotic entry in TT cells.

The distribution of cells in mitosis was evaluated in BHP7-13, WRO82-1 and 8505C cells (Figure 2D). Compared with control treatment, ganetespib significantly increased the percentage of prophase cells in BHP7-13 (97.0 ± 1.5% and 25.0 ± 4.0%, *P* < 0.001), prometaphase cells in WRO82-1 (70.0 ± 5.2% and 28.0 ± 3.3%, *P* < 0.001) and metaphase cells in 8505C (48.0 ± 5.9% and 23.0 ± 4.2%, *P* = 0.003), revealing mitotic arrest was in different mitotic phases.

The effect of ganetespib on the expression of cyclin-dependent kinase 1 (CDK1) was examined at 24 hours in BHP7-13, WRO82-1, 8505C and TT cells (Figure 2E). A low dose of ganetespib (6.25 nmol/L) was sufficient to reduce CDK1 expression in BHP7-13, WRO82-1 and 8505C. High doses of ganetespib (12.5 and 25 nmol/L) decreased CDK1 levels in four cell lines. Cell cycle transition from G2 to mitotic phase and mitotic progression from prophase to metaphase require increasing activity of CDK1-Cyclin B1 [20]. Ganetespib significantly represses CDK1 expression that may lead to G2 block and mitotic arrest in thyroid cancer cells. Our data are in line with ganetespib decreasing CDK1 expression in malignancies [13, 17].

### Modulation of proteins associated with RAS/RAF/ERK and PI3K/AKT/mTOR signaling by ganetespib

**Figure 3:**
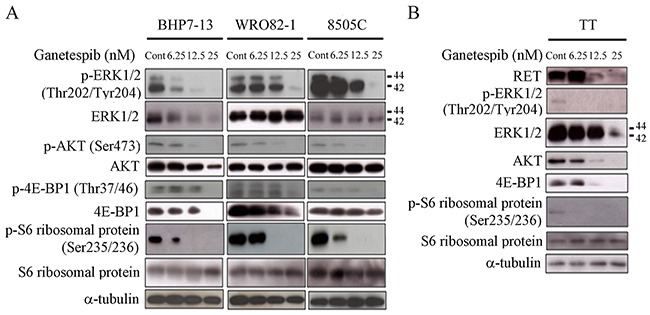
Ganetespib decreases expression of proteins involved in RAS/RAF/ERK and PI3K/AKT/mTOR signaling pathways and reduces RET level **(A)** The expression of phosphorylated and total ERK, AKT, 4E-BP1 and S6 ribosomal protein was evaluated using immunoblot in cells treated with ganetespib at indicated doses for 48 hours in BHP7-13, WRO82-1 and 8505C cells. Ganetespib decreased the levels of p-ERK1/2 (Thr202/Tyr204), p-AKT (Ser473), p-4E-BP1 (Thr37/46) and p-S6 ribosomal protein (Ser235/236) in a dose-dependent fashion in all three cell lines. Ganetespib also decreased the levels of ERK1/2, AKT and 4E-BP1 in BHP7-13, and 4E-BP1 in WRO82-1 with a dose-dependent manner. **(B)** The expression of RET, p-ERK1/2, ERK1/2, AKT, 4E-BP1, p-S6 ribosomal protein and S6 ribosomal protein were evaluated using Western blot in TT cells treated with ganetespib for 48 hours. Low dose of ganetespib (6.25 nmol/L) was sufficient to repress the expression of p-ERK1/2 and p-S6 ribosomal protein. High doses of ganetespib (12.5 and 25 nmol/L) reduced RET, AKT and 4E-BP1 levels. ERK1/2 expression was decreased by ganetespib at 25 nmol/L.

RAS/RAF/ERK and PI3K/AKT/mTOR signaling pathways are pivotal for the survival and growth of thyroid cancer cells [21, 22]. Ganetespib has been demonstrated to suppress proteins involved in these pathways [14–16]. Therefore, we evaluated the effects of ganetespib on the expression of phosphorylated and total ERK1/2, AKT, 4E-BP1 and S6 ribosomal protein at 48 hours in BHP7-13, WRO82-1 and 8505C (Figure 3A). Increasing doses of ganetespib led to greater decreases of p-ERK1/2 in BHP7-13 and 8505C, p-AKT and p-S6 ribosomal protein in BHP7-13, WRO82-1 and 8505C. A high dose of ganetespib (25 nmol/L) significantly decreased p-ERK1/2, p-AKT, p-4E-BP1 and p-S6 ribosomal protein in all three cell lines. This high dose of ganetespib (25 nmol/L) was also able to decrease the levels of total ERK1/2, AKT and 4E-BP1 in BHP7-13, and 4E-BP1 in WRO82-1.

We evaluated the effects of ganetespib on the expression of proteins associated with RAS/RAF/ERK and PI3K/AKT/mTOR pathways in the MTC cell line (TT) at 48 hours (Figure 3B). A low dose of ganetespib (6.25 nmol/L) was sufficient to reduce p-ERK1/2 and p-S6 ribosomal protein levels in TT cells. RET is a protooncoprotein that drives the survival and proliferation of TT cells. High doses of ganetespib (12.5 and 25 nmol/L) decreased RET, AKT and 4E-BP1 expression. Together, these data reveal ganetespib inhibits multiple pivotal survival and proliferation pathways in thyroid cancer cell lines.

### Effects of ganetespib on apoptosis

**Figure 4:**
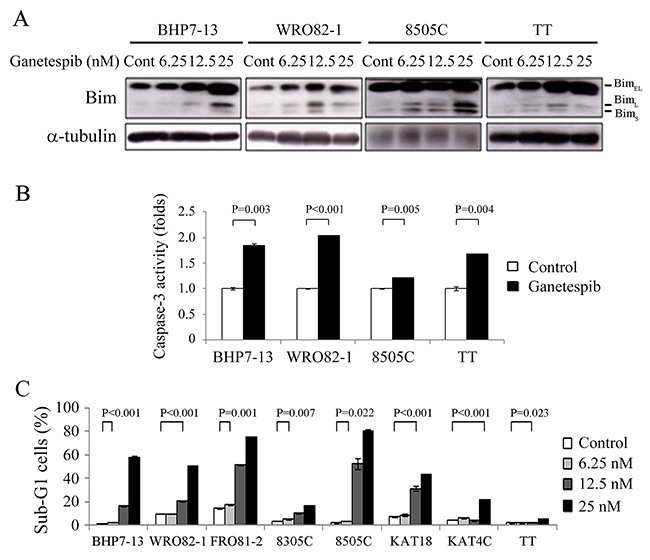
Ganetespib increases Bim expression, activates caspase-3 activity and induces apoptosis **(A)** Western blot was performed in cells treated with ganetespib for 48 hours in BHP7-13, WRO82-1, 8505C and TT. Ganetespib increased the expression of Bim isoforms in four cell lines. **(B)** Caspase-3 activity was detected using fluorometric assay kit in cells treated with ganetespib (25 nmol/L) or vehicle for 48 hours (BHP7-13, WRO82-1 and 8505C) and 72 hours (TT). **(C)** Sub-G1 apoptosis was detected by measuring DNA content using flow cytometry in cells treated with ganetespib for 72 hours. Ganetespib induced sub-G1 apoptotic cells in a dose-dependent manner in eight thyroid cancer cell lines.

The effect of ganetespib on Bim expression was evaluated at 48 hours in BHP7-13, WRO82-1, 8505C and TT cells (Figure 4A). Bim_EL_, Bim_L_ and Bim_S_ are three major isoforms of Bim that exhibit potent pro-apoptotic activity [23, 24]. Increasing doses of ganetespib induced higher expression of Bim_EL_ in BHP7-13, WRO82-1 and TT cells. Bim_L_ expression was induced in four cell lines. Ganetespib increased Bim_S_ level in WRO82-1 and 8505C cells.

The increased levels of Bim isoforms may lead to the activation of executioner caspase-3. The effects of ganetespib (25 nmol/L) on caspase-3 activity were determined using a fluorometric assay at 48 hours (BHP7-13, WRO82-1 and 8505C) and 72 hours (TT) (Figure 4B). Ganetespib significantly increased caspase-3 activity when compared with control in BHP7-13 (1.84 ± 0.04-fold and 1.00 ± 0.02-fold, *P* = 0.003), WRO82-1 (2.05 ± 0.01-fold and 1.00 ± 0.01-fold, *P* < 0.001), 8505C (1.21 ± 0.01-fold and 1.00 ± 0.01-fold, *P* = 0.005) and TT (1.68 ± 0.01-fold and 1.00 ± 0.04-fold, *P* = 0.004).

The activation of caspase-3 may result in apoptotic cell death. The ability of ganetespib to induce apoptosis in thyroid cancer cell lines was evaluated. Eight thyroid cancer lines were exposed to ganetespib for 72 hours and the proportions of sub-G1 apoptotic cells were calculated (Figure 4C). A low dose of ganetespib (6.25 nmol/L) significantly increased the proportion of sub-G1 cells in four cell lines (BHP7-13, FRO81-2, 8305C and 8505C). Ganetespib at 12.5 nmol/L meaningfully induced more sub-G1 cells in seven cell lines (BHP7-13, WRO82-1, FRO81-2, 8305C, 8505C, KAT18 and TT). A high dose of ganetespib (25 nmol/L) significantly increased the proportion of sub-G1 cells in all eight cell lines, demonstrating an induction of apoptosis.

### Ganetespib therapy of murine flank tumors

**Figure 5:**
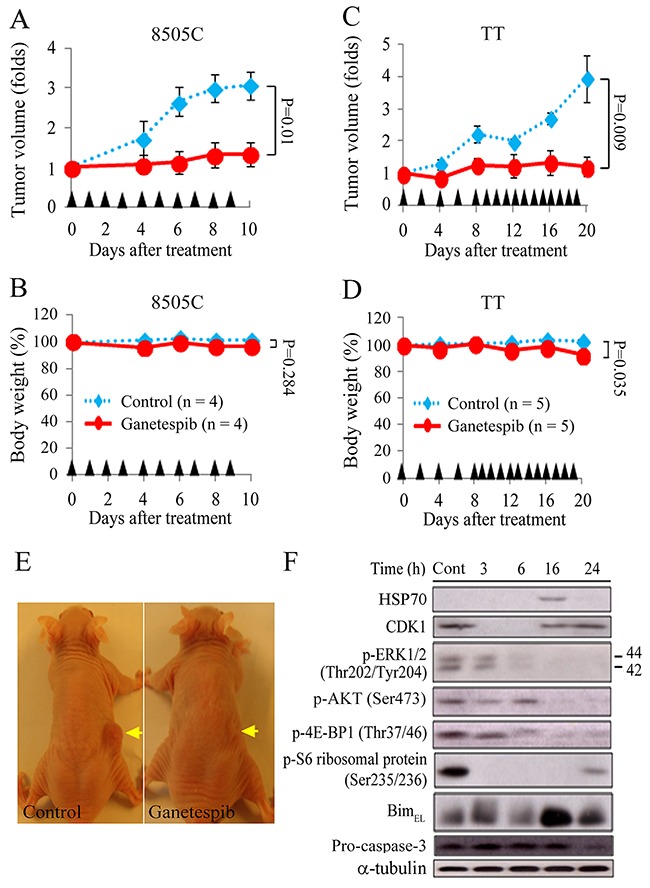
Ganetespib retards the growth of ATC and MTC flank xenograft tumors in nude mice **(A)** Compared with control tumors, daily intraperitoneal injection of ganetespib (50 mg/kg) significantly repressed 8505C tumor growth on day 6 (2.7 ± 0.4-fold and 1.1 ± 0.3-fold, *P* = 0.017), day 8 (3.0 ± 0.3-fold and 1.3 ± 0.3-fold, *P* = 0.011) and day 10 (3.1 ± 0.3-fold and 1.3 ± 0.3-fold, *P* = 0.01). **(B)** Sequential treatment of ganetespib did not significantly decrease weight in mice bearing 8505C tumors in comparison with control mice until day 10 (96.8 ± 2.9% and 101.4 ± 2.6%, *P* = 0.284). **(C)** Serial intraperitoneal injection of ganetespib (50 mg/kg) inhibited TT tumor growth. The differences in tumor growth between control and ganetespib groups reached statistical significance on day 16 (2.7 ± 0.2-fold and 1.3 ± 0.4-fold, *P* = 0.011) and day 20 (3.9 ± 0.7-fold and 1.2 ± 0.3-fold, *P* = 0.009). **(D)** Sequential treatment of ganetespib (50 mg/kg) slightly, but significantly induced weight loss in mice bearing TT tumors in comparison with control mice on day 20 (92.8 ± 2.0% and 102.3 ± 1.4%, *P* = 0.035). **(E)** Representative mice bearing 8505C tumors (arrows) were photographed at the conclusion of the study. **(F)** The molecular effects of single injection of ganetespib (50 mg/kg) in 8505C xenografts were evaluated using immunoblot. Arrowheads: intraperitoneal injections of placebo and ganetespib.

Athymic nude mice with flank xenografts of 8505C and TT were used to study the therapeutic efficacy and safety of ganetespib *in vivo*. The 8505C and TT cell lines were selected because they had a high tumorigenesis rate. Animals with established flank tumors reached mean diameters of 6.1 mm (8505C) and 4.7 mm (TT) were treated with serial intraperitoneal injections of ganetespib (50 mg/kg), a relevant dose in human cancer therapy [19]. Daily ganetespib treatment retarded 8505C tumor growth (Figure 5A). The difference in tumor volume increase between ganetespib and control mice reached statistical significance on day 6 (1.1 ± 0.3-fold and 2.7 ± 0.4-fold, *P* = 0.017) and the effect persisted through day 10 (1.3 ± 0.3-fold and 3.1 ± 0.3-fold, *P* = 0.01). Ganetespib did not significantly reduce body weight after a ten-day treatment in comparison with control mice (96.8 ± 2.9% and 101.4 ± 2.6%, *P* = 0.284; Figure 5B). Ganetespib also demonstrated therapeutic effects against TT tumors (Figure 5C). Serial treatment of ganetespib significantly retarded TT tumor growth on day 16 (1.3 ± 0.4-fold and 2.7 ± 0.2-fold, *P* = 0.011) and day 20 (1.2 ± 0.3-fold and 3.9 ± 0.7-fold, *P* = 0.009). Ganetespib significantly reduced body weight after a twenty-day treatment comparison with control mice (92.8 ± 2.0% and 102.3 ± 1.4%, *P* = 0.035; Figure 5D). Nevertheless, we did not observe any morbidity or decreased activity in these animals. The *in vivo* studies were closed when significant therapeutic effects persisted for 4 days. Representative mice bearing 8505C tumors were photographed when the study was closed (Figure 5E).

We evaluated the molecular effects of single ganetespib treatment on 8505C xenografts (Figure 5F). CDK1 was decreased between 3 to 6 hours. p-S6 ribosomal protein was repressed by 3 hours and the inhibitory effect persisted for 24 hours. p-ERK1/2 and p-4E-BP1 were decreased between 6 and 24 hours. p-AKT was decreased between 16 and 24 hours. Bim_EL_ was increased by 16 hours and the effect persisted for 24 hours. HSP70 expression was increased at 16 hours. Pro-caspase-3 level was decreased at 24 hours. These data suggested that ganetespib arrests cell cycle, inhibits RAS/RAF/ERK and PI3K/AKT/mTOR signaling pathways, and causes apoptosis *in vivo*.

## DISCUSSION

Ganetespib effectively inhibited cell proliferation in eight thyroid cancer lines originating from four major histologic types. A papillary thyroid cancer cell line (BHP7-13) represented the most sensitive cells. An ATC cell line (KAT18) was the most resistant. Overall, ganetespib had a relatively low median effect dose *in vitro* (≤ 8.5 nmol/L). Ganetespib effectively repressed tumor growth of relatively sensitive (8505C) and resistant (TT) thyroid cancer *in vivo*, suggesting that this agent has the potential for future clinical evaluations in the treatment of a range of thyroid malignancies. The gain of a compound with a novel molecular mechanism of activity against aggressive thyroid cancer may prove to have significant added clinical benefits.

Ganetespib inhibited mitotic entry and arrested mitotic progression in thyroid cancer cells. CDK1-Cyclin B1 is pivotal to trigger mitotic entry and increasing the activity of this complex leads to mitotic progression from prophase to metaphase [20]. A reduction of CDK1 expression may cause a failure in G2 to mitosis transition and arrest in mitotic progression in thyroid cancer cell lines.

The four major histologic types of thyroid cancer consist of different oncogenic alterations. The development of PTC and FTC are primarily driven by genetic alterations either in the RAS/RAF/ERK pathway or PI3K/AKT/mTOR pathway. Full activation of both pathways resulting from accumulated genetic alterations in both signaling cascades leads to the development of ATC [21]. For MTC, *RET* protooncogene occurs in almost all familial cases (25% of MTC) and about 40% of cases of sporadic MTC [25]. The oncogenic protein RET leads to the activation of RAS/RAF/ERK and PI3K/AKT/mTOR signaling. In sporadic MTC without *RET* mutations, 69.2% of tumor samples show *RAS* mutations that can activate RAS/RAF/ERK and PI3K/AKT/mTOR pathways [26, 27]. In this study, ganetespib inhibited RAS/RAF/ERK and PI3K/AKT/mTOR signaling in four histologic types of thyroid cancer cell lines and decreased RET level in a MTC cell line. The inhibitory effects on multiple signaling cascades may lead to the promising therapeutic efficacy of ganetespib [28]. The correlations between ganetespib sensitivity and decreases in CDK1 levels, or decreases in phosphorylated or total levels of ERK1/2, AKT, 4E-BP1, S6 ribosomal protein and RET in eight thyroid cancer cell lines were not specifically evaluated in this study.

Ganetespib increased Bim, activated caspase-3 and induced apoptosis, demonstrating that ganetespib induces apoptotic cell death in thyroid cancer. The expression of Bim is partly regulated by the PI3K/AKT/mTOR and RAS/RAF/ERK pathways [29]. Inhibition of PI3K/AKT signaling increases the transcription of Bim. The inactivation of ERK1/2 prevents the degradation of Bim. Ganetespib inhibited both signaling pathways which likely contributed to the increased expression of Bim that may subsequently activate caspase-3 and induce apoptosis in thyroid cancer cells.

Ganetespib treatment significantly inhibited 8505C and TT tumor growth with favorable safety profiles in this study. In 8505C xenografts, CDK1 level was decreased, which may lead to cell cycle arrest in G2/M phase. The decreased levels of p-ERK1/2, p-AKT, p-4E-BP1 and p-S6 ribosomal protein suggested that RAS/RAF/ERK and PI3K/AKT/mTOR signaling pathways were inhibited. The Bim_EL_ level was increased between 16 and 24 hours, which may activate caspase-3 activity during this period. However, pro-caspase-3 was decreased only at 24 hours, not at 16 hours. The overexpression of HSP70 at 16 hours may inhibit caspase-3 degradation and prevent apoptosis [30]. Simultaneous inhibition of HSP90 and HSP70 may provide a better therapeutic effect than HSP90 inhibition alone in the treatment of ATC [31].

We also evaluated the molecular effects of ganetespib therapy in TT tumors (Supplementary Figure 2). As expected, the expression of CDK1, p-ERK1/2 and p-S6 ribosomal protein was decreased. However, RET, AKT and p-4E-BP1 levels were not decreased and Bim_EL_ expression was not increased. These data indicate ganetespib inhibited TT tumor growth through decreasing CDK1, p-ERK1/2 and p-S6 ribosomal protein levels.

WRO82-1 and 8505C cells harbor oncogenic *BRAF*^V600E^ mutation [32, 33]. Ganetespib decreases the expression of mutant BRAF^V600E^ in melanoma cells [34]. We found ganetespib decreased BRAF levels in WRO82-1 and 8505C cell lines (Supplementary Figure 3). Therefore, this inhibitory effect may be one of the mechanisms contributing to cytotoxicity in these two cell lines.

In conclusion, ganetespib effectively induced cytotoxicity in four major pathologic types of thyroid cancer. Nude mice bearing ATC and MTC xenograft tumors proved the therapeutic efficacy and safety of ganetespib. These data encourage future clinical trials studying the utility of ganetespib to treat patients with thyroid cancer.

## MATERIALS AND METHODS

### Cell lines

Eight cell lines were evaluated, including a papillary (BHP7-13), a follicular (WRO82-1), a follicular undifferentiated (FRO81-2), four anaplastic (8305C, 8505C, KAT18 and KAT4C) and a medullary (TT) human thyroid cancer cell lines. All cell lines except KAT4C were authenticated using DNA short tandem repeats profiling and stored in liquid nitrogen until use [33]. BHP7-13, WRO82-1, FRO81-2, KAT18 and KAT4C were maintained in RPMI 1640 with sodium bicarbonate (2.0 g/L). 8305C and 8505C were maintained in MEM with sodium pyruvate (1 mmol/L) and sodium bicarbonate (2.2 g/L). TT was maintained in F12K. All media contained 10% FCS, 100,000 units/L penicillin and 100 mg/L streptomycin. All cells were maintained in a 5% CO_2_ humidified incubator at 37°C.

### Pharmacologic agents

Ganetespib was obtained from Selleck Chemicals. Ganetespib (10 mmol/L) was dissolved in DMSO (Sigma) and stored at -30°C until *in vitro* experiments. For *in vivo* studies, ganetespib (5.5 mg/ml) was diluted before use with a solution that contained 10% DMSO, 18% Cremophor RH 40 (Sigma), 3.6% dextrose (Sigma) and 68.4% water.

### Antibodies

The antibodies targeting HSP70, p-ERK1/2 (Thr202/Tyr204), ERK1/2, p-AKT (Ser473), AKT, p-4E-BP1 (Thr37/46), 4E-BP1, p-S6 ribosomal protein (Ser235/236), S6 ribosomal protein, RET, Bim and pro-caspase-3 were from Cell Signaling Technology. CDK1 and α-tubulin antibodies were from Sigma.

### Cytotoxicity assays

Cells were plated at 2 × 10^4^ (TT) or 2 × 10^3^ cells (other cell lines) per well in 24-well plates in 1 mL media. After overnight incubation, six serial 1:1 dilutions of ganetespib or vehicle were added at the starting dose of 100 nmol/L over a 4-day treatment course. Cytotoxicity was determined on day 4. Cells were washed with PBS and lysed with Triton X-100 (1.35%, Sigma) to release intracellular lactate dehydrogenase (LDH), which was quantified with a Cytotox 96 kit (Promega) at 490 nM by spectrophotometry (Infinite M200 PRO, Tecan). The results were shown as the percentage of surviving cells determined by comparing the LDH activity of each sample to that of control samples which were considered 100% viable. Median effect doses (Dm) on day 4 were calculated for each cell line using CompuSyn software [35, 36].

### Western blots

Cells were plated at 1 × 10^6^ cells per 100-mm Petri dish in 10 mL media overnight and treated with ganetespib or vehicle for indicated periods. Cell pellets were dissolved in a radio-immunoprecipitation assay buffer with a protease inhibitor cocktail, vortexed and clarified by centrifugation. Total protein (10-20 μg) was electrophoresed on 10-12% Tris-HCl gels, transferred to polyvinylidene difluoride membranes, blocked, and exposed to primary antibodies followed by a secondary antibody conjugated to horseradish peroxidase. Signals were developed using an enhanced chemiluminescence kit (PerkinElmer).

### Cell cycle assessment

To evaluate the effects of ganetespib on cell cycle progression, cells were plated at 4 × 10^4^ (KAT4C), 5 × 10^4^ (8305C and TT) or 1 × 10^5^ cells (BHP7-13, WRO82-1, FRO81-2, 8505C and KAT18) per well in 6-well plates in 2 mL media overnight. Ganetespib or vehicle was added at indicated doses for 48 hours (TT) or 24 hours (other cell lines). Adherent cells were trypsinized, washed with PBS, fixed with cold 70% ethanol and incubated with RNase A (100 μg/mL; Sigma) and propidium iodide (PI, 5 μg/mL; Sigma) at 37°C for 15 minutes. The cell cycle distribution was assessed by DNA content detected by flow cytometry (BD FACScalibur Flow Cytometer, BD Biosciences). Each condition was performed in triplicate.

The effect of ganetespib on mitotic progression was evaluated using confocal microscopy. Thyroid cancer cells were plated at 5 × 10^4^ cells in four-well culture slides in 1 mL of media overnight. Cells were treated with ganetespib (25 nmol/L) or placebo for 48 hours (TT) or 24 hours (other cell lines), washed with PBS, fixed in 4% paraformaldehyde (Sigma) for 15 minutes at room temperature, washed with PBS, permeabilized with 0.1% Triton X-100 (10 minutes, room temperature), washed with PBS, incubated with primary mouse α-tubulin antibody (1:1000) at 4°C overnight, washed with PBS and incubated with secondary AlexaFluor 488-conjugated goat anti-mouse antibody (1:1000; Life Technologies) for 25 minutes at 37°C, washed with PBS, counterstained with 4’,6-diamidino-2-phenylindole (DAPI; 0.2 μg/mL, Invitrogen), washed with PBS and covered with mounting media. Images were acquired using Leica TCS SP8 X confocal microscopy. Chromosomes were examined to identify mitotic cells.

### Apoptosis analyses

Caspase-3 activity was analyzed using fluorometric assay kit (Abcam). Cells were plated at 1 × 10^6^ cells in 100-mm Petri dishes in 10 mL of media overnight. Ganetespib (25 nmol/L) or vehicle was added for 72 hours (TT) or 48 hours (other cell lines). Adherent cells (5 × 10^5^) were collected, centrifuged, lysed using 50 μL of lysis buffer on ice for 10 minutes, incubated with DEVD-AFC substrate and reaction buffer at 37°C for 1.5 hour. Caspase-3 activity was detected by spectrophotometry. The fluorescence intensity of the treated samples was compared with that of control sample to determine the fold-increase in caspase-3 activity. Each condition was performed in duplicate.

The ability of ganetespib to induce sub-G1 apoptotic cells was studied using flow cytometry. Cells were plated at 4 × 10^4^ (KAT4C) or 1 × 10^5^ cells (other cell lines) per well in 6-well plates in 2 mL media overnight. Ganetespib was added at indicated doses. Floating cells and trypsinized adherent cells were collected at 72 hours and samples were prepared as described above for cell cycle analysis. Apoptotic sub-G1 cells were detected by measuring DNA content using flow cytometry (BD FACScalibur Flow Cytometer, BD Biosciences). Each condition was performed in triplicate.

### Flank xenograft tumor therapy

Six-week-old athymic female nude mice (National Laboratory Animal Center, Taiwan) were anesthetized by intraperitoneal injection of 2% 2,2,2-tribromoethanol (200 μl/mouse; Sigma) before implantation of thyroid cancer cells. 8505C and TT flank tumors were established by injecting 1 × 10^6^ cells in 100 μL of ECM gel (Sigma) subcutaneously into the flank of nude mice. When 8505C tumors reached 6.1 mm in mean diameter (*n* = 4 per group), the mice received daily intraperitoneal injections of ganetespib (50 mg/kg) or placebo. Mice with TT xenografts (mean diameter 4.7 mm, *n* = 5 per group) were treated with intraperitoneal injections of ganetespib (50 mg/kg) or placebo every 2 days for 5 injections, followed by daily treatment. Tumor dimensions were serially measured with electronic calipers, and tumor volume was calculated by the formula of a × b^2^ × 0.4, where a represents the greatest diameter and b is the perpendicular diameter. The body weight and physical activity of each animal were followed as markers of toxicity.

Tumor levels of HSP70, CDK1, p-ERK1/2 (Thr202/Tyr204), p-AKT (Ser473), p-4E-BP1 (Thr37/46), p-S6 ribosomal protein (Ser235/236), Bim and pro-caspase-3 were evaluated in mice treated with a single dose of ganetespib (50 mg/kg). At indicated periods, the animals were euthanized with carbon dioxide and tumors were harvested, mixed with protein extraction buffer (GE Healthcare), homogenized and sonicated on ice. After centrifugation, clarified supernatants were aliquoted and stored at -20°C for western blotting.

This study was performed in accordance with the recommendations of the Guide for the Care and Use of Laboratory Animals of Chang Gung Memorial Hospital and the protocol was approved by the Committee of Laboratory Animal Center at Chang Gung Memorial Hospital, Linkou (permission No: 2012120301).

### Statistical analyses

Comparison was performed when appropriate using two-sided Student's *t* test (Excel, Microsoft). *P* < 0.05 was considered statistically significant. The results were expressed as mean ± SE.

## SUPPLEMENTARY MATERIALS FIGURES


