# Supplementary material for: Efficacy of an HSP90 inhibitor, ganetespib, in preclinical thyroid cancer models
Source: Oncotarget. 2017 Apr 18;8(25):41294–304. doi: 10.18632/oncotarget.17180 (PMC5522292; doi:10.18632/oncotarget.17180)
Supplement: Supplementary file 1 [file oncotarget-08-41294-s001.pdf]

Efficacy of an HSP90 inhibitor, ganetespib, in preclinical thyroid cancer models

SUPPLEMENTARY MATERIALS

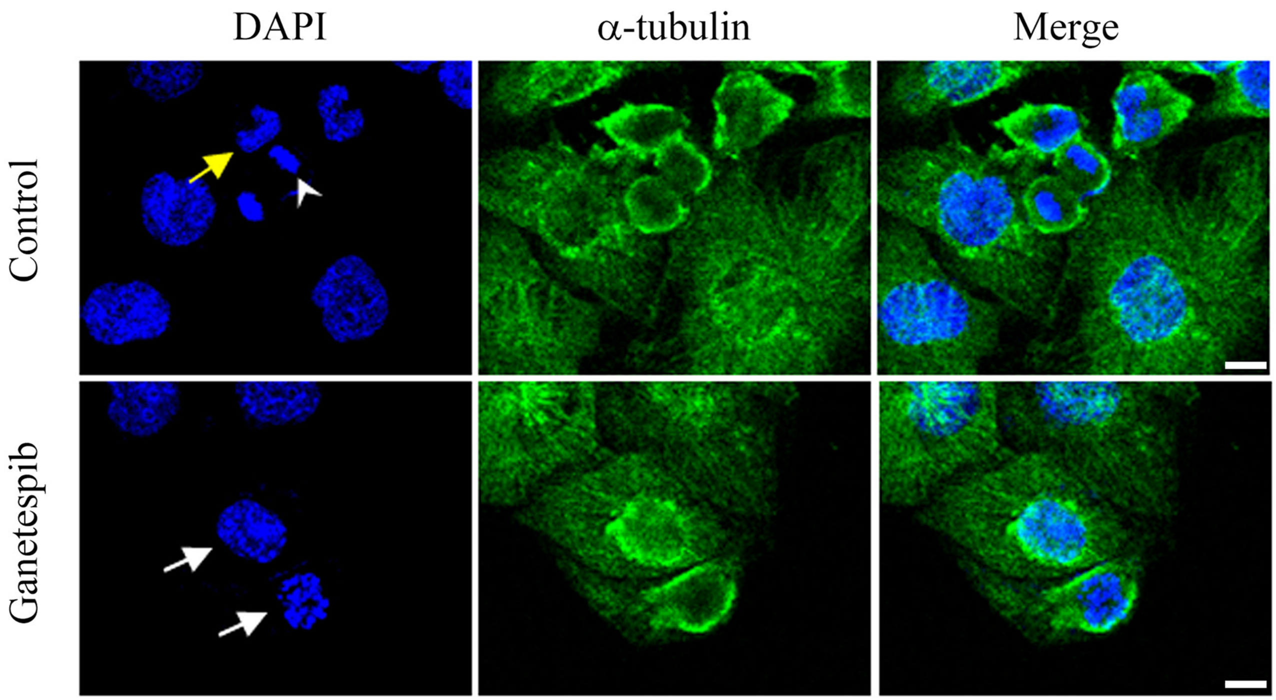

Supplementary Figure 1: The effect of ganetespib on mitosis in BHP7-13 cells. Chromosomal appearance was evaluated in BHP7-13 cells treated with ganetespib (25 nmol/L) or placebo for 24 hours using immunofluorescence confocal microscopy. Cells were stained with DAPI and α-tubulin. Placebo-treated cells at prometaphase (yellow arrow) and anaphase (white arrowhead) were indicated. Ganetespib-treated cells at prophase (white arrows) were demonstrated. Scale bar, 10 μm.

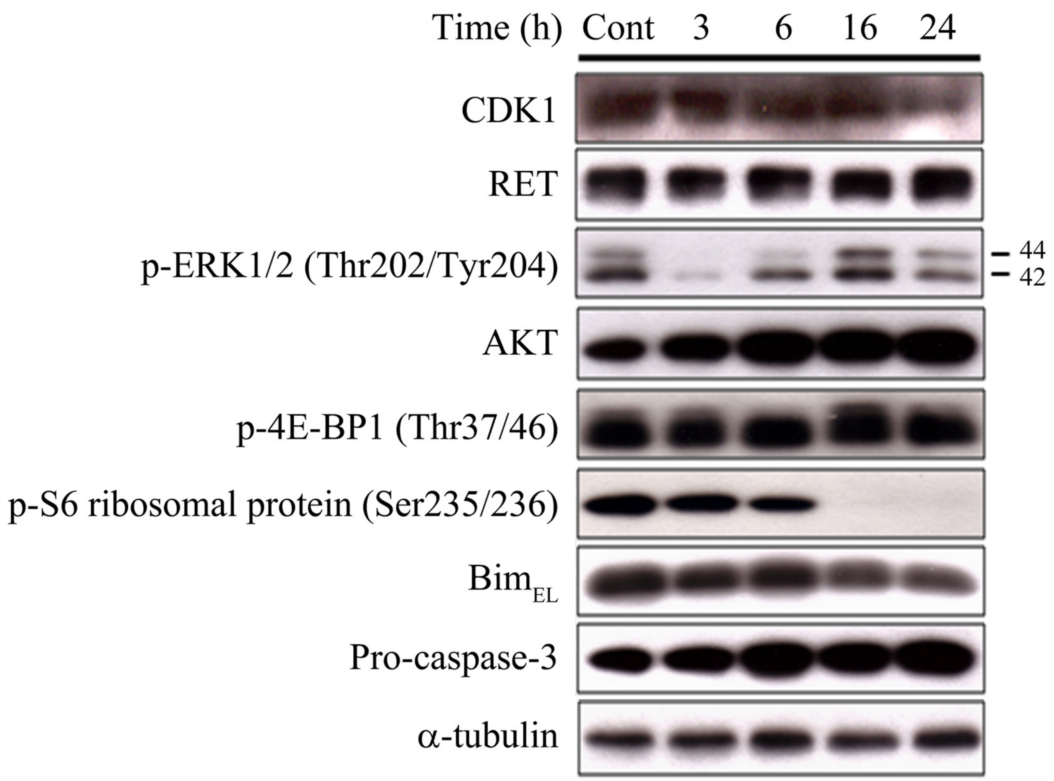

Supplementary Figure 2: The effects of ganetespib treatment in TT tumors. The molecular effects of single injection of ganetespib (50 mg/kg) in TT xenografts were evaluated using immunoblot. CDK1 level was decreased by 24 hours. p-ERK1/2 level was decreased at 3 hours. p-S6 ribosomal protein and Bim_{EL} levels were decreased by 16 hours and the effect persisted for 24 hours. AKT and pro-caspase-3 levels were increased between 6 and 24 hours. RET and p-4E-BP1 levels were not obviously changed.

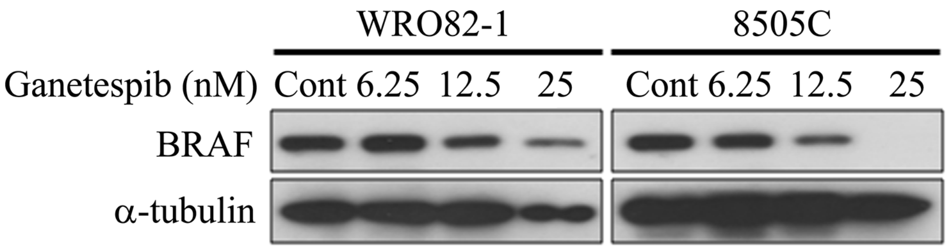

Supplementary Figure 3: Ganetespib decreases BRAF expression in WRO82-1 and 8505C cell lines. The expression of BRAF was evaluated using immunoblot in cells treated with ganetespib at indicated doses for 48 hours in WRO82-1 and 8505C cells. Ganetespib decreased BRAF levels in a dose-dependent fashion in these cell lines.
